# Real-World Patterns and Decision Drivers of Radiotherapy for Lung Cancer Patients in Romania: RADIO-NET Study Results

**DOI:** 10.3390/diagnostics12123089

**Published:** 2022-12-08

**Authors:** Mihai-Teodor Georgescu, Renata Zahu, Petronela Rusu, Gabriela Teodorescu, Gabriel Kacso

**Affiliations:** 1 Department of Oncology, “Carol Davila” University of Medicine and Pharmacy, 050474 Bucharest, Romania; 2 “Prof. Dr. Alexandru Trestioreanu” Oncology Institute, 022328 Bucharest, Romania; 3 Radiotherapy Center Amethyst Cluj-Napoca, 407280 Cluj, Romania; 4 Department of Oncology-Radiotherapy, “Iuliu Hatieganu” University of Medicine and Pharmacy Cluj-Napoca, 400012 Cluj, Romania; 5 “Prof. Dr. Ion Chiricuta” Oncology Institute Cluj-Napoca, 400015 Cluj, Romania; 6 AstraZeneca, 013713 Bucharest, Romania

**Keywords:** lung cancer, real-world, radiotherapy, curative intent, palliation

## Abstract

Radiotherapy (RT) plays a crucial role in all stages of lung cancer. Data on recent real-world RT patterns and main drivers of RT decisions in lung cancer in Romania is scarce; we aimed to address these knowledge gaps through this physician-led medical chart review in 16 RT centers across the country. Consecutive patients with lung cancer receiving RT as part of their disease management between May–October 2019 (pre-COVID-19 pandemic) were included. Descriptive statistics were generated for all variables. This cohort included 422 patients: median age 63 years, males 76%, stages I–II 6%, III 43%, IV 50%, mostly adeno- and squamous cell carcinoma (76%), ECOG 0-1 50% at the time of RT. Curative intent RT was used in 36% of cases, palliative RT in 64%. Delays were reported in 13% of patients, mostly due to machine breakdown (67%). Most acute reported RT toxicity was esophagitis (19%). Multiple disease-, patient-, physician- and context-related drivers counted in the decision-making process. This is the first detailed analysis of RT use in lung cancer in Romania. Palliative RT still dominates the landscape. Earlier diagnosis, coordinated multidisciplinary strategies, and the true impact of the multimodal treatments on survival are strongly needed to improve lung cancer outcomes.

## 1. Introduction

Lung cancer is a major healthcare problem, being the worldwide leading cause of oncological mortality despite significant progresses made in the last decade in diagnosis and treatment modalities [1,2]. In Romania also lung cancer is ranked first as incidence (12% of all new cases) and cancer-related mortality (20% of all deaths) in 2020 [3].

The management of lung cancer is complex and includes multimodal treatments, with radiotherapy (RT) playing a crucial role across all stages of the disease, in curative or palliative setting [4,5]. The rapid advances in imaging technologies and delivery systems contribute to the dynamism of the RT landscape and allow a more targeted approach and improved patient outcomes [5,6,7]. However, to what extent and how fast these advances can be applied in routine cancer care remains unknown. Previous studies have shown that RT is under-utilized in clinical practice, which negatively impacts the patient’s survival [1,8,9]. The need for radiation therapy for new lung cancer cases is projected to increase globally with 18% and in Romania with 14% by 2025 as compared to 2012 [10]. Disparities and variations in cancer management including RT across different healthcare systems and hospitals within the same country or region have been previously described [1,6,11,12,13,14,15].

Real-world insights are very informative for clinicians and policymakers since patient populations are more heterogenous compared to those included in randomized clinical trials [16,17]. Recent real-world evidence derived from various lung cancer cohorts in different geographies indicates wide diagnostic and treatment patterns in lung cancer [9,17,18,19,20], thus emphasizing the need to better understand the local context and to learn from other centers or countries’ best practices to improve outcomes. Romania lacks a national cancer registry and the latest epidemiology trends are based only on data collected by the cancer registries from the north-western part of the country (affiliated with the European Network of Cancer Registries) [21,22]. In consequence, information on treatment patterns used in clinical practice in lung cancer across the country is scarce. For this reason, and as a first step to generate real-world evidence across the country, we performed this retrospective, observational study focused on RT patterns and use in lung cancer patients. In addition, this study explored the parameters of the decision-making process used by radiation oncologists for their lung cancer patients.

## 2. Materials and Methods

### 2.1. Study Design

The “Real world patterns and rationale of the RADIOtherapy in lung caNcEr paTients from Romania” (RADIO-NET) was a national retrospective, non-interventional, physician-led medical chart review. Sixteen public and private RT clinics participated in the RADIO-NET study. 

Study investigators were radiation oncologists in charge of RT decisions of lung cancer at each study center. The Investigators reviewed the medical records of patients with lung cancer, who received RT between May-October 2019 as part of their disease management. In addition, each site provided site-specific information about existing RT facilities. All data were collected in an anonymized way in a secured web-based data capture system. 

The study was approved by the National Committee of Bioethics for Medicines and Medical Products, with a waiver of informed consent (15SNI/3 November 2020) and complied with the Declaration of Helsinki and regulations of the participating institutions.

### 2.2. Study Objectives

This study had two primary objectives: to describe RT patterns of use in lung cancer patients and to characterize the main criteria used by physicians in establishing the type of used RT.

The secondary objectives were to describe the demographic and clinical characteristics of the patients included and the relationships with the delivered RT. There were no defined survival endpoints.

### 2.3. Study Population

Eligible patients were adults (age ≥ 18 years) with a confirmed diagnosis of lung cancer who received RT during a pre-defined study period of 6 months. Patients were identified in the reverse chronological order of delivered RT, starting with October 2019. The site’s quota was 30 patients for the 6-month period of interest, except for cases when a site identified >30 patients eligible in October 2019. In such cases, sites were allowed to enroll all patients eligible to receive RT in October 2019. In case the site’s quota was not reached during October 2019, the chart review continued in the previous month for a maximum of 6 months (until the quota was reached or May 2019). 

The only exclusion criterion was the concurrent participation of the patient in a clinical trial at the time of RT administration. 

### 2.4. Variables

The variables collected were part of the general oncological and radiological assessments and management of lung cancer patients per routine clinical practice based on national or institutional protocols and guidelines in use in 2019. 

Primary variables included radiation treatment characteristics (such as type of RT, site of irradiation, dosage, treatment planning, image guiding, delineation protocol, organs at risk, sequence as to the systemic therapy, RT actual delivery, and toxicities) and patient-, disease-, physician-related or contextual criteria used by physicians to guide the RT decision. Secondary variables included demographic information and clinical parameters. 

### 2.5. Statistical Analysis

Sample size was defined based on the feasibility information registered from the RT centers, considering the number of patients managed with RT in the study-defined period. No formal statistical hypotheses were set. Statistical analyses were descriptive. Analyses were performed on datasets collected from all eligible patients in the entire cohort and on two groups stratified by type of RT received (curative RT and palliative RT, based on treatment intent). All patients enrolled who met all inclusion criteria, without fulfilling the exclusion criterion formed the full analysis set (FAS). 

The statistical summaries included frequencies and proportions for categorical variables, and mean, standard deviation, median, and range for continuous variables. Correlations between the type of RT and clinical characteristics of patients (age, gender, histological type of tumor, tumor stage, duration of cancer) were assessed by means of various statistical tests (e.g., chi-squared, Student, Mann–Whitney–Wilcoxon). The relationships between the variables were explored through regression analysis. Two-sided 95% confidence intervals (CI) of the frequencies and means were provided. The significance level was set at *p* < 0.05. The statistical analyses were performed using version 4.0.2 of the *R* package (https://www.r-project.org/, accessed on 1 March 2021).

## 3. Results

### 3.1. Site Characteristics

Out of the 16 RT centers participating in this study, 5 (31%) were regional or national cancer institutes, 8 (50%) private hospitals, 1 (6%) university hospital, 1 (6%) general hospital and 1 (6%) private university hospital. Most centers provided integrated oncology care (n = 14, 88%), while the other 2 (12%) provided exclusively RT services.

All sites had linear accelerators with various upgrades, with intensity modulated radiation therapy (IMRT) in 11 (69%) sites, including volumetric modulated arc therapy (VMAT) in 10 (63%) sites. Stereotactic ability was present in four (25%) sites and brachytherapy technology in only one (6%) site. Other characteristics such as on-board imaging and set-up correction protocol are described in Table 1.

The average RT time slot/patient had a median (range) of 13.5 (9–20) minutes, with a minimum RT time slot/patient of 10 (5–15) minutes and a maximum RT time slot/patient of 30 (10–60) minutes. 

### 3.2. Patient Characteristics

In total, 427 medical charts were reviewed by study investigators; 5 cases had no confirmed lung cancer diagnosis at the time of applying RT (emergency scenario) and were excluded from the analysis. Hence, the FAS included 422 patients, out of which 36% received curative RT, and the rest (64%) palliative RT. Detailed patient characteristics in the FAS and study groups are presented in Table 2. In the FAS, most patients were male (76%), with a median age at the time of initial lung cancer diagnosis of 63 years, and similar age and gender characteristics in study groups. At the time of the irradiation treatment, one-third of patients (33%) from the FAS were current smokers. 

In the FAS, the most frequent histological diagnosis was adenocarcinoma (49%), followed by squamous cell carcinoma (27%), with a significantly different distribution across study groups (curative versus palliative RT) and a small size effect (*p* = 0.002, _ωCohen_ = 0.24). In the FAS, the most common tumor stage at diagnosis was metastatic (50%), with a significantly different distribution across study groups with a large size effect (*p* < 0.001, _δCliff_ = 0.68). 

### 3.3. RT Characteristics

As shown in Table 3, treatment approaches were heterogenous, with VMAT and three-dimensional conformal radiation therapy (3D-CRT) being the most frequently reported treatment approaches used (40% and 35%, respectively). The delineation protocols used in the curative group included in all cases the planning target volume (PTV), and distinct clinical target volume (CTV) of the primary tumor and lymph nodes (88% and 85%, respectively), derived from the gross tumor volume (GTV) counterparts. In the palliative RT group, the PTV only (for example, whole brain RT) was the main delineation protocol used (78%) (Supplemental Table S1). The distribution of organs at risk (OARs) in study groups and palliative RT subgroups varied based on the site of irradiation (Supplemental Table S2). 

Treatment delays were registered for one-tenth of patients (13%) overall, and these were due in general to machine breakdown. Acute toxicities were reported for one-fourth of all patients (26%), with more in the curative RT group (46%). In the curative RT group, radiation esophagitis was reported for most patients experiencing acute toxicities (93%). 

In the group with curative intent (n = 152), definitive RT was applied most frequently (73%), followed by adjuvant RT (18%) and neo-adjuvant RT (9%). The most often used dose in the curative RT group was 54-60 Gy standard fractionation (47%); the remainder received either <54 Gy (28%) or >60 Gy (25%). The minimum dose applied was 10 Gy in the neo-adjuvant setting and the maximum dose was 66.6 Gy. Most patients in the curative setting group received 11-30 fractions (63%) or >30 fractions (28%), while the rest received <10 fractions (9%). For most patients in the curative RT group (88%), nodal irradiation was reported. 

In the group with palliative RT (n = 270), the most common doses were 30 Gy/10 fractions (43%) and 20 Gy/5 fractions (32%). The sites of irradiation in the palliative group were whole brain (50%), bone (27%), and thorax (23%), which included primary tumors, mediastinal lymph nodes, or pleuro-pulmonary metastases.

#### Chemoradiotherapy and Other Treatments 

Chemoradiation (CRT) was administered during the studied period in two-thirds of patients from the curative RT group (n = 100 [66%]) and in less than one-fourth from the palliative RT group (n = 60 [22%]). In general, sequential CRT was used (57% in the curative group and 92% in the palliative group). Overall, the most frequent chemotherapy agents used in patients receiving CRT (n = 160; pooled data) were carboplatin (61%), cisplatin (31%), etoposide (26%), paclitaxel (21%), and vinorelbine (18%) (Supplemental Table S3). 

For almost one-third of all patients (n = 129 [31%]) pre-RT treatments consisting of targeted therapy, immunotherapy, and/or surgery were reported (n = 44 [29%] in the curative RT and n = 85 [32%] in the palliative group). Almost 40% of all patients had other treatments (primarily chemotherapy and immunotherapy) reported up to 6 months after the RT, although missing information on post-RT treatments was reported for 43% of patients across the study (Supplemental Table S4). 

### 3.4. Drivers of RT Decision 

**Figure 1:**
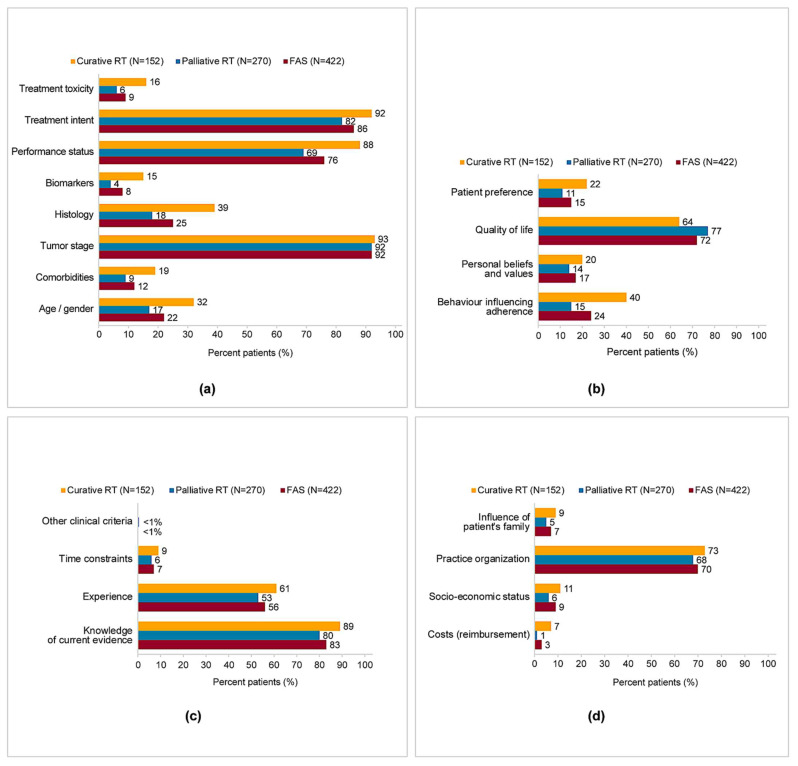
The drivers of medical decision to select the specific RT regimen: (**a**) disease-related criteria; (**b**) patient-related criteria; (**c**) physician-related criteria; (**d**) context-related criteria. Note: multiple responses per patient.

Across the FAS, the most frequent disease-related criteria considered by physicians at the time of deciding the RT regimen were tumor stage (92%) and patient’s performance status (76%), with relatively similar proportions in study groups (Figure 1a). 

The main patient-related criterion considered at the time of deciding the RT regimen was quality of life (72%), numerically higher for the patients in the palliative RT group (77%) versus curative RT (64%), whereas patient’s behavior potentially influencing adherence was a factor considered mostly in patients from the curative RT group (40%) versus palliative RT (15%) (Figure 1b). For a proportion of 17% of patients across FAS and in each study group, no patient-related criterion relevant to the decision was reported. 

Knowledge of medical evidence (83%) and radiation oncologist’s experience (56%) were the main physician-related criteria taken into account at the time of the RT decision, with similar proportions in study groups (Figure 1c). 

The most frequently reported contextual criterion considered at the time of deciding the RT regimen was related to the practice organization (70%), with similar rates in study groups (Figure 1d). In some patients, prolonged overall treatment time, machine breakdown and holidays were listed among the criteria impacting the treatment regimen (overall rates ≤ 1%). In one-fifth of the entire cases (26%) no contextual criterion was considered relevant for the RT decision. 

### 3.5. Regression Models

The logistic regression models indicated that tumor stage and performance status were significant factors influencing the treatment intent, with the likelihood to receive RT with palliative intent increasing as the tumor stage was more advanced and/or the performance status was poorer. 

The first model included only tumor stage as the independent variable (OR = 37.3, 95% CI: 20.3–68.9, β = 3.6, *pseudo R^2^_McFadden_* = 0.5), and the second model both tumor stage and performance status (OR = 28.5, 95% CI: 15.3–53.3, β = 3.4 for tumor stage, and OR = 2.9, 95% CI: 1.8–3B2; = 1.1 for performance status, *pseudo R^2^_McFadden_* = 0.6). However, the second model showed only a small increase in the explained deviance over the first model, as shown by the goodness of fit indicator the *pseudo R^2^_McFadden_*. No other variables produced viable logistic models. 

## 4. Discussion

To our knowledge, this is the first centralized analysis of recent radiation treatment patterns and drivers of the decision to select specific RT regimens in real-life settings in patients with lung cancer from Romania. Over a 6-month interval in 2019, the palliative RT dominated in a consecutive, unselected sample of 422 patients with lung cancer, being used in almost two-thirds of patients. The analysis of treatment decision drivers illustrated its complexity and multi-dimensional nature. These results provide a snapshot of the local RT use in lung cancer and will serve as a baseline for future studies. As such, our analysis should be seen as an instrument to self-reflect and enhance the collaboration among the professional oncology groups in our country, with a fast track from lung cancer diagnosis to up-to-date staging and multidisciplinary tumor board decision. 

This study was conducted in 16 RT centers across the country, equally distributed between public and private practices, with one-third of regional/national cancer institutes. Most centers (88%) offered integrated oncology care, and two were exclusive radiotherapy units. While the pillars of decision-making were similar across centers, high heterogeneity of RT protocols was observed. Variations in the RT use and regimens may be explained by patient-, disease- and physician-related factors that usually build the multi-layered oncological treatment decision [14,23], but also the local infrastructure and type of center [20]. In at least half of the centers, modern RT techniques (VMAT, IMRT, and IGRT) were available, proving real technological progress as compared to the ESTRO-HERO survey [24] conducted in 2014 in Southern and Central Eastern Europe countries. Yet, even though the number of linear accelerators has doubled in Romania in the past 5 years, the regional coverage remains disproportionate and suboptimal in many geographical areas [25], a result in line with the general under-utilization of RT [1,8,9]. 

The RADIO-NET patient population seems younger than other large real-life cohorts (median age at diagnosis 63 years vs. 66 or 70 years) [16,26]. Age at diagnosis may be influenced by national health policies, with median age being reported lower in countries where elderly patients do not benefit of healthcare programs with a comprehensive range of investigations [27]. More than half of patients (61%) in our study presented with metastatic stage at the time of initial diagnosis. In line with this finding and data reported in the literature, palliation radiotherapy was still more frequently applied (64% of patients in this group) [28,29]. Heterogenous by number and localization, distant metastases are important prognostic factors [30], and the metastatic disease has poor survival [31,32]. Therefore, active steps to increase lung cancer detection in earlier stages are needed at all levels. 

Public and private partnerships and alliances prove useful in identifying the needs across geographies, aligning the possible approaches in lung cancer screening, and calling to action to fight cancer at a high level [33,34]. The low dose computed tomography screening has demonstrated benefits in reducing lung cancer mortality and detecting earlier-stage disease in clinical trials in high-risk populations [35,36,37]. However, implementation in real-life requires adequate infrastructure and workforce and may be more problematic [38]. Specifically for Romania, preliminary measures such as opportunistic screening for smokers might be simpler and faster to implement at a larger scale if specific guidelines are put in place [39]. Another direction to improve is the staging process, which is currently hampered by delays in the diagnosis flow and sub-optimal access to imaging services. The intra- and post-pandemic boost of telemedicine and artificial intelligence might alleviate these bottlenecks. The national cancer control plan was recently launched for discussions, and it aims to reduce by 25% the proportion of lung cancers diagnosed in late stage and to pilot a screening program for lung cancer [40]. 

In our set, curative RT was applied to one-third of patients (36%). The rapid technological advances in the RT field allow now for higher doses ranging from 60 to 70 Gy in the curative setting of lung cancer, with the maximum tolerable dose being limited by the application to the organ at risk (OARs). Increasing the PTV dose even >70 Gy is possible as long as the dosimetry constraints to the OAR are respected [5,41,42], despite the negative, yet criticized, results of the RTOG 0617 trial [43]. In our curative RT group, more than half of patients (61%) received RT doses ≥60 Gy, the maximum dose being 66.6 Gy. CRT was received by two-thirds (≈66%) of patients in the curative RT group, with sequential administration in more than half of these cases, despite current recommendations favoring concurrent CRT given its statistically significant superior median 3- and 5-year overall survival benefit of around 5% [41,44]. The literature cites interstitial lung abnormalities, severe chronic obstructive diseases, poor performance status, and increased risk for radiation esophagitis as factors influencing the decision of how CRT is administered [4,45]. In our clinical practice, patients’ refusal of chemotherapy, delays of RT start (due to infrastructure deficit), and absence of integrated medical oncology facilities in some RT centers may be additional causes of the lack of concomitant CRT. We consider this low percentage of concomitant CRT in curative intent setting alarming, calling for more detailed discussions at the local level.

Acute toxicities were reported for almost half of patients from the RT curative group (46%) and much less in the palliative group (15%). A recent meta-analysis of RT toxicities in non-small cell lung cancer (NSCLC) showed that the highest toxicity is described for concurrent CRT, followed by sequential CRT, and curative RT alone, with the lowest in the palliative setting [46]. The advances in RT techniques with improved delivery and treatment planning ensure now better results, with lower rates of toxicities and higher precision [47,48,49]. Of particular importance are the 4D-CT acquisition for motion management and the systematic PET-CT fusion target volumes delineation, able to significantly reduce the main OARs relevant exposure. Our results show that 4D-CT and/or PET-CT fusion for treatment planning was obviously underused due to a lack of 4D-CT infrastructure, and bureaucratic delays in obtaining a free-of-charge PET-CT, requiring centralized approval. Our findings suggest an under-evaluation of toxicities, which might be partially explained by the fractured care of patients in many centers, and the lack of standardized reporting of radiation toxicities in routine clinical practice. A more formal and harmonized organization of patient care delivery is urgently needed at the local and national levels to improve the workflow at all stages and across all components of multimodal management. 

The second primary aim of this study was to understand the drivers of treatment decisions made by radiation oncologists for their patients with lung cancer. In this pathology, numerous factors related to the disease, patient, physician, healthcare system, or infrastructure, besides the geographic variations of RT practice are intricated in treatment decisions [50,51,52]. The weight of all factors influencing the decision-making process is different and varies considerably among organizations and even experts [20,23]. As expected, the treatment intent correlated significantly (*p* < 0.001) with tumor stage alone and both tumor stage and performance status Although the specific details of the target volume and OARs delineation were not collected, the reported disparities in contouring GTV, CTV, and PTV should trigger a national consensus meeting and more strict institutional guidelines with internal and external specific audits on RT’s minimum mandatory technical requirements, outside the acknowledged interobserver variability

With a minimal set of inclusion and exclusion criteria, this study gathered nationwide data in a centralized manner, thus building the first real-world evidence on RT patterns in Romania in a stable period, before the coronavirus disease 2019 (COVID-19) outbreak. COVID-19 impacted severely the healthcare settings and oncology services at all levels: diagnosis, treatment, and symptom palliation. Analyses of RT use during the first wave of COVID-19 showed that the diagnostic work-up and treatments were altered, radical RT reduced, hypofractionated regimens increased and palliative care was deferred or sometimes never performed [53,54,55]. This is the main reason why we considered the year 2019 more relevant for providing a reference snapshot of the RT patterns across Romania. Nevertheless, the observational, retrospective design of this study, the short period of data collection, and the site and patient sampling process limited the generalizability of results. Data collection relied entirely on the information existing in the medical charts, which is not standardized and prone to missing or insufficient data. The RT-related data were available and accurate, but the pre- and post-RT disease history and treatment details were limited or missing in patients’ files in RT departments. In many centers, the RT and medical oncology records are kept separately and, in general, patients visit the RT centers for specialized treatment and then return to the local oncology network. Thus, the RT centers lack complete data on further treatments, patient outcomes, or vital status. This fragmented care hindered all oncological treatments description and survival analyses. Although we explored the relationship between the type of RT received and clinical characteristics, no causality can be proven due to multiple confounding factors. 

Our real-life analysis improved knowledge about current radiation treatment patterns and drivers of RT decisions in patients with indications for irradiation as part of their lung cancer management in Romania. On a different note, it emphasized that data sharing, communication, and coordination between all oncology specialists are fundamental to improving earlier diagnosis and care delivery. Our data will assist our national healthcare providers and professional societies involved in lung cancer diagnosis and therapy to better understand, collaborate, and solve the areas of unmet needs at the institutional and national levels. 

## Figures and Tables

**Table 1:** Site facilities.

Characteristic	N = 16 n (%)
Linear accelerator upgrades	
Intensity modulated radiation therapy	11 (69)
Volumetric modulated arc therapy	10 (63)
Image-guided radiation therapy	9 (56)
Multi-leaf collimator (MLC)	8 (50)
Micro-MLC	8 (50)
On-board imaging (OBI)	
kV OBI	13 (81)
Cone beam computed tomography	12 (75)
Electronic portal imaging device	8 (50)
Megavoltage computed tomography	4 (25)
Set-up correction protocols	
Extended no action level (eNAL)	7 (44)
No action level	4 (25)
Shrinking action level	4 (25)
Prescription isodose level	3 (19)
Daily imaging	2 (13)
On-line correction	2 (13)

Note: These are pooled data (multiple responses).

**Table 2:** Patient characteristics in the overall set, curative RT and palliative RT groups.

Characteristic	FAS (N = 422)	Curative RT (N = 152)	Palliative RT (N = 270)
Age at the time of initial lung cancer diagnosis, median (min–max), years	63 (27–87)	64 (27–83)	64 (31–87)
Age at the time of receiving RT, median (min–max), years	64 (27–87)	64 (27–83)	64 (33–87)
Males, n (%)	322 (76)	120 (79)	202 (75)
Smoking history, n (%)			
Active smokers	140 (33)	63 (41)	77 (29)
Ex-smokers	102 (24)	35 (23)	67 (25)
Non-smokers	43 (10)	14 (9)	29 (11)
Unknown	137 (33)	40 (26)	97 (36)
Comorbidities (reported rate > 5%)			
Chronic obstructive pulmonary disease	142 (34)	48 (32)	94 (35)
Heart failure	39 (9)	14 (9)	25 (9)
Diabetes mellitus	39 (9)	11 (7)	28 (10)
Gastro-intestinal disorders	28 (7)	6 (4)	22 (8)
Cerebrovascular disease	24 (6)	6 (4)	18 (7)
Other types of cancer ^1^	26 (6)	15 (10)	11 (4)
ECOG performance score at the time of RT, n (%)			
0–1	211 (50)	117 (77)	94 (35)
≥2	204 (48)	35 (23)	169 (63)
Unknown	7 (2)	-	7 (3)
*p*-value between groups ^2^	-	<0.001	
Duration of disease at the time of RT, median (min–max), years (since initial diagnosis)	0.4 (0–12.9)	0.3 (0.1–2.5)	0.4 (0–12.9)
Tumor histology, n (%)			
Adenocarcinoma	205 (49)	55 (36)	150 (56)
Squamous cell carcinoma	112 (27)	59 (39)	53 (20)
Small cell carcinoma	77 (18)	28 (17)	49 (18)
Other histological type ^3^	28 (7)	10 (7)	18 (7)
*p*-value between groups ^4^	-	0.002	
Tumor stage at the time of initial lung cancer diagnosis, n (%)			
Early stage (IA-IIB)	23 (6)	14 (9)	9 (3)
Limited stage (IIIA)	48 (11)	40 (26)	8 (3)
Locally advanced (IIIB-IIIC)	134 (32)	84 (55)	50 (19)
Metastatic (IV)	211 (50)	14 (9)	197 (73)
Unknown	6 (1)	0 (0)	6 (2)
*p*-value between groups ^5^	-	<0.001	
Genetic mutational testing performed, n (%)	180 (43)	63 (41)	117 (43)
Positive *EGFR* mutation ^6^	34 (19)	8 (13)	26 (22)
Positive PD-L1 expression ^6^	72 (40)	34 (54)	38 (33)

^1^ Other types of cancer included most frequently urothelial cancers, breast cancers, and colorectal cancers; ^2^ significance tested using Mann–Whitney–Wilcoxon test; a large effect size was identified (δ_Cliff_ = 0.49); ^3^ other histological types included large cell carcinomas, mixed histology, sarcomatoid carcinoma and lung cancer not otherwise specified; ^4^ significance tested using Chi-squared test, χ^2^ = 24.16, df = 5; a small effect size was identified (ω_Cohen_ = 0.24); ^5^ significance tested using Mann–Whitney–Wilcoxon test; a large effect size was identified (δ_Cliff_ = 0.68); ^6^ expressed out of the total number of patients with genetic testing performed across the FAS (n = 180) and in each study group, respectively (n = 63 in the curative RT group, and n = 117 in the palliative RT group). Note: due to rounding, percentages may not be always 100%. Abbreviations: ECOG = Eastern Cooperative Oncology Group, EGFR = epidermal growth factor receptor, PD-L1 = programmed death-ligand 1, RT = radiotherapy.

**Table 3:** RT characteristics in the overall set, curative, and palliative RT groups.

RT Characteristics	FAS (N = 422)	Curative RT (N = 152)	Palliative RT (N = 270)
Treatment planning and image guidance ^1^, n (%)			
IMRT non-VMAT	59 (14)	23 (15)	36 (13)
FDG PET-CT fusion	22 (5)	21 (7)	1 (<1)
VMAT tomotherapy	30 (7)	9 (5)	21 (8)
VMAT non-tomotherapy	154 (36)	94 (62)	60 (22)
3D-CRT	147 (35)	28 (18)	119 (44)
4D-CT	2 (<1)	2 (1)	-
2D	11 (3)	2 (1)	9 (3)
SRS and SBRT	9 (2)	2 (1)	7 (3)
Delay characteristics			
Patients experiencing delays, n (%)	54 (13)	33 (22)	21 (8)
Duration of delay, median (min–max), days	3 (1–12)	4 (2–12)	3 (1–10)
Main causes of RT delay ^2^, n (%)			
Radiation toxicity	8 (15)	7 (21)	1 (5)
Machine breakdown	36 (67)	25 (76)	11 (52)
Technical revision	10 (19)	9 (27)	1 (5)
Patient-related factors	9 (17)	6 (18)	3 (14)
Other ^3^	10 (20)	2 (6)	8 (38)
Acute toxicities			
Patients experiencing acute RT toxicities, n (%)	110 (26)	70 (46)	40 (15)
Type of acute RT toxicities ^4^, n (%)			
Esophagitis	78 (71)	65 (93)	13 (33)
Pneumonitis	16 (15)	15 (21)	1 (3)
Skin toxicity	20 (18)	16 (23)	4 (10)
Neurotoxicity	23 (21)	-	23 (58)

^1^ Data inconclusive for 17 (6%) patients in the palliative RT group; ^2^ expressed out of the total number of patients experiencing delays in the FAS (n = 54), the curative RT group (n = 22), and the palliative group (n = 21), respectively; multiple reasons could have been provided for one case; ^3^ other included intercurrent illnesses, hypoglycemia, holidays, and treatment start in another RT center; ^4^ expressed out of the total number of patients with acute toxicities reported in the FAS (n = 110), and the curative RT group (n = 70) and palliative group (n = 40), respectively. Note: multiple responses per patient. Abbreviations: 2D = two-dimensional, 3D-CRT = three-dimensional conformal radiation therapy, 4D-CT = four-dimensional computed-tomography; FAS = full analysis set, FDG PET-CT = fluorodeoxyglucose-positron emission tomography, IMRT = image-modulated radiation therapy, RT = radiotherapy, SBRT = stereotactic body radiation, SRS = stereotactic radiosurgery, VMAT = volumetric modulated arc therapy.

## Data Availability

The data presented in this study may be provided upon reasonable request to the study sponsor.

## Supplementary Materials

The following supporting information can be downloaded at: https://www.mdpi.com/article/10.3390/diagnostics12123089/s1, Table S1: Distribution of delineation protocols in the overall set, curative and palliative RT groups (multiple responses); Table S2: Distribution of organs at risk (OARs) in the overall set, curative and palliative RT groups (multiple responses); Table S3: Agents used during chemoradiation (including targeted and immunotherapy in combination with chemotherapy regimens) in the overall set, curative and palliative RT groups (pooled data, multiple responses); Table S4: Post-RT treatment classes in the overall set, curative and palliative RT groups (pooled data, multiple responses).
