# Supplementary material for: Real-World Patterns and Decision Drivers of Radiotherapy for Lung Cancer Patients in Romania: RADIO-NET Study Results
Source: Diagnostics (Basel). 2022 Dec 8;12(12):3089. doi: 10.3390/diagnostics12123089 (PMC9777374; doi:10.3390/diagnostics12123089)
Supplement: Supplementary file 1 [file diagnostics-12-03089-s001.zip › diagnostics-2041343-supplementary.pdf]

Table S1. Distribution of delineation protocols in the overall set, curative, and palliative RT groups (multiple responses)

Delineation protocol, n (%)	FAS (N=422)	Curative RT (N=152)	Palliative RT (N=270)
Gross tumor volume (GTV) of the primary tumor	172 (41)	106 (70)	66 (24)
GTV of the lymph nodes	99 (24)	74 (49)	25 (9)
Clinical target volume (CTV) of the primary tumor	209 (50)	133 (88)	76 (28)
CTV of the lymph nodes	150 (36)	129 (85)	21 (8)
Planning target volume	363 (86)	152 (100)	211 (78)
Internal target volume (ITV)	35 (8)	30 (20)	5 (2)
Mid-ventilation/mid-position approaches	16 (4)	14 (9)	2 (1)
Other	120 (28)	4 (3)	116 (43)
Whole brain	76 (18)	3 (2)	73 (27)
Bone metastases	41 (10)	0	41 (15)
Bronchial stump	1 (<1)	1 (<1)	0
Pleural metastases	1 (<1)	0	1 (<1)
Spinal cord/kidneys/liver/lungs	1 (<1)	0	1 (<1)

Table S2. Distribution of organs at risk (OARs) in the overall set, curative, and palliative RT groups (multiple responses)

OARs, n (%)	FAS (N=422)	Curative RT (N=152)	Palliative RT (N=270)
Spinal cord[1]	291 (69)	146 (96)	145 (54)
Lungs[1]	226 (54)	144 (95)	82 (30)
Heart[1]	218 (52)	143 (94)	75 (28)
Oesophagus[1]	205 (49)	135 (89)	70 (26)
Brachial plexus	34 (8)	26 (17)	8 (3)
Trachea and proximal bronchial tree	46 (11)	31 (20)	15 (6)
Other (reported rates >10%)			
Lens	121 (29)	5 (3)	116 (43)
Eye globes	61 (15)	3 (2)	58 (22)
Optic chiasm	59 (14)	0	59 (22)
Brainstem	55 (13)	0	55 (21)
Optic nerves	55 (13)	0	55 (21)
None	24 (6)	0	24 (9)

[1]Data inconclusive for a number of 6 (4%) patients in the curative RT group.

Table S3. Agents used during chemoradiation (including targeted and immunotherapy in combination with chemotherapy regimens) in the overall set, curative, and palliative RT groups (pooled data, multiple responses)

Chemotherapy agents, n (%)	FAS (N=160)	Curative RT (N=100)	Palliative RT (N=60)
Carboplatin	98 (61)	63 (63)	35 (58)
Cisplatin	49 (31)	32 (3)	17 (28)
Docetaxel	3 (2)	1 (1)	2 (3)
Etoposide	42 (26)	22 (22)	20 (33)
Gemcitabine	22 (14)	11 (11)	11 (18)
Paclitaxel	24 (21)	25 (25)	9 (15)
Albumin-bound paclitaxel	1 (<1)	1 (1)	0
Pemetrexed	15 (9)	3 (3)	12 (20)

Vincristine	1 (<1)	0	1 (2)
Vinorelbine	29 (18)	23 (23)	6 (10)
Vinblastine	1 (<1)	1 (1)	0
Other			
Bevacizumab	2 (1)	0	2 (3)
Irinotecan	1 (<1)	1 (1)	0
Chemoimmunotherapy with pembrolizumab and paclitaxel	1 (<1)	1 (1)	0
Unknown	3 (2)	2 (2)	1 (2)

Table S4. Post-RT treatment classes in the overall set, curative, and palliative RT groups (pooled data, multiple responses)

**Post-RT treatment classes, n (%)**	**FAS (N=164)**	**Curative RT (N=50)**	**Palliative RT (N=114)**
Targeted therapy	12 (7)	3 (6)	9 (8)
Immune therapy	64 (39)	21 (42)	43 (38)
Surgery	3 (2)	2 (4)	1 (1)
Other therapy	107 (65)	32 (64)	75 (66)
Chemotherapy	78 (48)	16 (32)	62 (54)
Radiotherapy	23 (14)	14 (28)	9 (8)
Osteoclast inhibitors	9 (6)	1 (2.0)	8 (7)
Chemoimmunotherapy	2 (1)	2 (4)	0
Chemoimmunotherapy & radiotherapy	1 (<1)	1 (2)	0
Immunotherapy	1 (<1)	0	1 (1)
Radiotherapy & immunotherapy	1 (<1)	0	1 (1)
Other[1]	3 (2)	1 (2)	2 (2)

[1]Other included symptomatic therapy (non-specified), pleurodesis and opioids.
